# Images in Vascular Medicine: Peripheral artery thrombosis in critically ill patients with COVID-19

**DOI:** 10.1177/1358863X20938431

**Published:** 2020-07-15

**Authors:** Nuri Tutar, Burcu Baran Ketencioglu, Şahin Temel, Kürşat Gündoğan, Özgür Karabıyık, Murat Sungur

**Affiliations:** 1 Department of Pulmonary Medicine, School of Medicine, Erciyes University, Kayseri, Turkey; 2 Department of Internal Medicine, Division of Intensive Care Unit, School of Medicine, Erciyes University, Kayseri, Turkey; 3 Department of Radiology, School of Medicine, Erciyes University, Kayseri, Turkey

A 75-year-old man with a history of hypertension and type 2 diabetes mellitus presented to the coronavirus disease 2019 (COVID-19) outpatient clinic with a sore throat and cough for 5 days. History was negative for coronary artery disease and he was not taking antiplatelet or anticoagulation therapy on admission. A physical exam was notable for fever (36.9°C, 103.3°F), blood pressure (BP) 120/70 mmHg, and oxygen saturation (SpO_2_) 96% on room air without tachypnea. His blood examination showed C-reactive protein (CRP) was 91.7 mg/L (normal range: 0–5 mg/L), white blood cell count (WBC) was 9310/mm^3^ (4800–10,700/mm^3^) with 1400/mm^3^ lymphocytes (1300–2900/mm^3^), and a platelet count was 261 × 10^9^/L (130–400 × 10^9^/L). His chest computed tomography (CT) revealed bilateral ground glass opacities with peripheral and basilar predominance (Panel A-1); nasopharyngeal polymerase chain reaction (PCR) testing for COVID-19 was positive. The patient was hospitalized and started on hydroxychloroquine, levofloxacin, and oseltamivir. His D-dimer was 1630 ng/mL (0–500 ng/mL) and enoxaparin sodium 40 mg once a day was started. On the 5th day of hospitalization, his oxygen requirements increased and the patient was transferred to the intensive care unit (ICU). Favipiravir and piperacillin-tazobactam were started with proning protocols. The patient progressed to SpO_2_ 70% on 100% FiO_2_ with a non-rebreather mask (NRB) and was eventually intubated. Five days later, darkening and ischemic changes in the right toes developed, consistent with gangrene (Panel A-2). Doppler ultrasound revealed thrombosis in the right great saphenous vein and deep crural veins below the knee; in addition, there was no flow in the right distal dorsalis pedis artery. We did not perform CT angiography because of renal insufficiency.

**Panel A.**
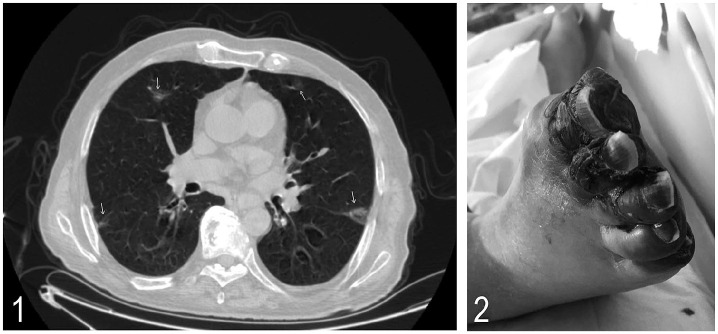


A second patient, a 77-year-old woman with a history of hypertension, type 2 diabetes mellitus, coronary artery disease, and cerebrovascular disease (on clopidogrel monotherapy), was also admitted to the COVID-19 outpatient clinic with fever. Vital signs at presentation were temperature 39.1°C, heart rate 115 bpm, BP 160/80 mmHg, respiration rate (RR) 22, SpO_2_ 96% on room air. A blood test showed WBC: 16,000/mm^3^, lymphocytes: 800/mm^3^, platelet count: 261 × 10^9^/L, and CRP: 128 mg/L. The D-dimer level was not obtained at the time of admission. Nasopharyngeal PCR testing for COVID-19 was positive despite a normal-appearing chest CT on admission, consistent with the early phase of this disease. The patient was hospitalized and started on hydroxychloroquine and piperacillin-tazobactam. The patient was transferred to the ICU on day 2 of hospitalization due to worsening respiratory status (RR 30, PaO_2_/FiO_2_ ratio 200). Favipiravir and unfractionated heparin (UFH) 5000 IU Q 8 hours subcutaneously were added and proning protocols were initiated. The D-dimer level was 6900 ng/mL and her chest X-ray showed bilateral infiltrates (Panel B-1). The patient was intubated because her PaO_2_ was 52 mmHg on 100% FiO_2_. On the second day of intubation, her right leg became red and cold (Panel B-3). A hypodense filling defect in the right proximal superficial femoral artery was observed (Panel B-2, arrow) and the left superficial femoral artery appeared normal on review of her CT angiography images (Panel B-2, dotted arrow). The occlusion caused by the filling defect was demonstrated clearly on an anterior-view volume-rendering CT angiogram (Panel B-4, arrow), and, on the opposite side, normal continuity of the superficial femoral artery was detected (Panel B-4, dotted arrow). A Doppler ultrasound for the venous system was normal.

**Panel B.**
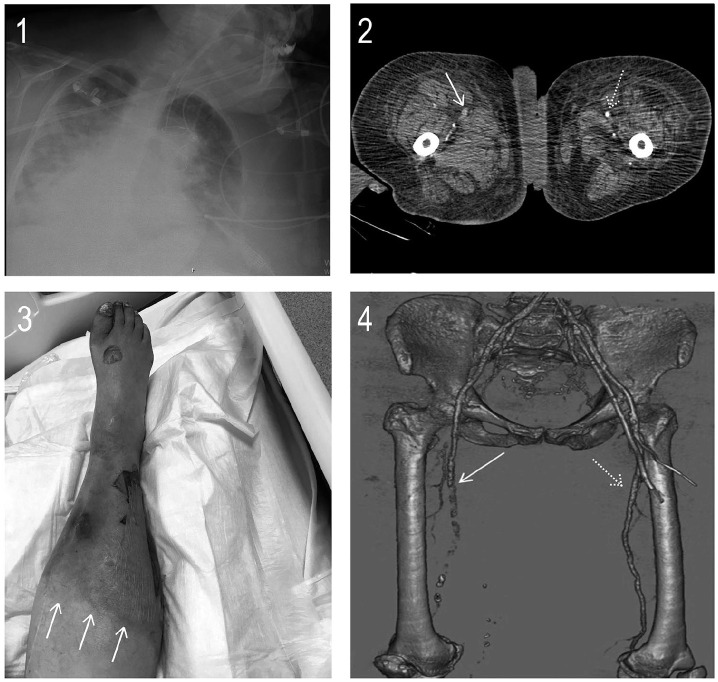


Both patients were treated with UFH (80 units/kg bolus and 18 units/kg/h infusion intravenously). The activated partial thromboplastin time (aPTT) reached therapeutic range (55–88 seconds) after administration of UFH and no heparin-induced thrombocytopenia findings were detected during follow-up. Atrial fibrillation (AF) was not detected during the course of follow-up for the first patient; the second patient experienced AF several days after the arterial occlusion was detected. Both patients received vasopressor therapy days after the onset of their arterial occlusions due to worsening shock, and ultimately expired on the 12th and 14th days of hospitalization, respectively.

COVID-19 may display a variable course in different populations around the world, and the reported mortality differs across various geographies.^1^ The reported frequency of venous thromboembolism (VTE) is 25% in patients with severe COVID-19 in China who did not receive VTE prophylaxis; however, VTE was identified in up to 69% of European patients receiving prophylactic and treatment dose anticoagulation.^2,3^ The largest publication of patients with COVID-19 admitted to the ICU (184 patients) demonstrated a cumulative frequency of thrombotic events in 31%: 25 of the 31 cases were due to pulmonary embolism, three were deep vein thrombosis, and three were arterial events (ischemic stroke).^4^ Acute peripheral thrombosis has also been reported in severely ill patients with COVID-19 from Spain, some of which describe cutaneous findings similar to our patients.^5,6^ Interestingly, an Italian series of 384 patients (61 ICU patients) did not report any arterial thrombotic events.^7^

To conclude, COVID-19 is a disease that can be seen with thrombotic complications. It is important to note that both arterial and venous thrombosis can occur. The development of these complications while on thromboprophylaxis suggests that increased anticoagulant doses may be needed for severely ill patients. The newly published British Thoracic Society guideline emphasizes the use of intermediate dose anticoagulants for high-risk patients (i.e. enoxaparin 40 mg twice daily),^8^ although this is an area of active investigation and some providers are empirically treating with full dose anticoagulation.^9^

‘Images in vascular medicine’ is a regular feature of *Vascular Medicine*. Readers may submit original, unpublished images related to clinical vascular medicine. Submissions may be sent to: Heather Gornik, Editor in Chief, *Vascular Medicine*, via the web-based submission system at http://mc.manuscriptcentral.com/vascular-medicine
